# Phosphoproteomics and Lung Cancer Research

**DOI:** 10.3390/ijms131012287

**Published:** 2012-09-26

**Authors:** Elena López, William C. S. Cho

**Affiliations:** 1 Hospital Universitario Niño Jesús, Department of Oncohematology of Children, Madrid 28009, Spain; E-Mail: elena.lopez.villar@gmail.com; 2 Department of Clinical Oncology, Queen Elizabeth Hospital, Hong Kong

**Keywords:** lung cancer, mass spectrometry, phosphoproteomics, post-translational modification, signaling pathway

## Abstract

Massive evidence suggests that genetic abnormalities contribute to the development of lung cancer. These molecular abnormalities may serve as diagnostic, prognostic and predictive biomarkers for this deadly disease. It is imperative to search these biomarkers in different tumorigenesis pathways so as to provide the most appropriate therapy for each individual patient with lung malignancy. Phosphoproteomics is a promising technology for the identification of biomarkers and novel therapeutic targets for cancer. Thousands of proteins interact via physical and chemical association. Moreover, some proteins can covalently modify other proteins post-translationally. These post-translational modifications ultimately give rise to the emergent functions of cells in sequence, space and time. Phosphoproteomics clinical researches imply the comprehensive analysis of the proteins that are expressed in cells or tissues and can be employed at different stages. In addition, understanding the functions of phosphorylated proteins requires the study of proteomes as linked systems rather than collections of individual protein molecules. In fact, proteomics approaches coupled with affinity chromatography strategies followed by mass spectrometry have been used to elucidate relevant biological questions. This article will discuss the relevant clues of post-translational modifications, phosphorylated proteins, and useful proteomics approaches to identify molecular cancer signatures. The recent progress in phosphoproteomics research in lung cancer will be also discussed.

## 1. Introduction

Lung cancer is the most commonly diagnosed cancer and the leading cause of cancer-related death worldwide [1]. Massive evidence suggests that genetic abnormalities contribute to the development of lung cancer. These molecular abnormalities may serve as diagnostic, prognostic and predictive biomarkers for this deadly disease. It is imperative to search these biomarkers in different tumorigenesis pathways so as to provide the most appropriate therapy for each individual patient with lung malignancy. Proteomics is a promising technology for the identification of biomarkers and novel therapeutic targets for cancer [2]. Previous proteomics studies have shown a number of biomarkers discovered in lung cancer, such as serum amyloid A, isocitrate dehydrogenase 1 and karyopherin alpha 2 [3–5]. Recent advances in phosphoproteomics technology also enable the identification of biomarkers by proteome phosphorylation analysis in a variety of scenarios that can be implemented in biomedical research and translational setting [6].

Phosphoproteomics includes methods and tools that enable research including, but not limited to, instrumentation, techniques, devices and analysis tools. The identification and definition of the molecular profiles of cancer require the development and dissemination of high-throughput molecular analysis technologies as well as elucidation of all of the molecular species embedded in the genome and proteome of cancer and normal cells. Moreover, the main challenge in cancer control and prevention is the early detection of cancer. This will enable effective interventions and therapies contributing to the reduction in mortality and morbidity. At a specific time, biomarkers serve as molecular signposts of the physiologic state of a cell. These signposts are the result of genes, their products (proteins) and other organic chemicals made by the cell. Biomarkers prove to be vital for the identification of early cancer and those subjects at risk of developing cancer as a normal cell progresses through the complex process of transformation to a cancerous state [7–14]. This article will discuss the ongoing researches in post-translational modifications (PTMs), phosphorylated proteins, and useful proteomics approaches to identify molecular cancer signatures. The recent progress in phosphoproteomics research in lung cancer will be also discussed.

### 1.1. PTMs

Many proteins are post-translationally modified and it is those modifications that often govern the functions of these proteins. PTM is the covalent attachment of a chemical group to a protein after protein synthesis or the proteolytical removal of a signal peptide after localization. Proteins, which are either membrane-bound or designed to be secreted, carry a signal sequence known as a signal peptide. This signal sequence is usually positioned at the *N*-terminal part of the protein, but in some proteins the signal sequence is an internal sequence. These signal peptides direct the newly synthesized proteins into the endoplasmatic reticulum in preparation for secretion from the cell [15]. *N*-terminal signal sequences are removed after translocation and are therefore rarely found in mature proteins. The attachment of a chemical group can change the protein structure, the affinity for a given target molecule, the chemical properties and thereby considerably alter the activities of a protein. Within the modifications involved in cellular regulation and signaling, many are reversible processes, wherein the shift between the addition and removal of the modifying group provides highly controlled regulation. There are many different kinds of PTMs. The addition of poly-ubiquitin to a protein by an ubiquitinylation enzyme complex targets proteins for degradation by the proteasome [16]. Methylation of histone H3 catalyzed by a methyl transferase CARM1 has been shown to stimulate transcription [17]. In addition, phosphorylation catalyzed by protein kinases and dephosphorylation catalyzed by protein phosphatases are central in numerous cellular processes. A small number, but central PTMs are illustrated in Table 1.

### 1.2. Phosphorylated Proteins

Phosphorylation is a common PTM in proteins. Genomic sequencing has shown that around 4% of all eukaryotic genes are likely to code for a protein kinase: the catalyst of phosphorylation [28]. It has also been suggested that one third of all proteins being expressed in a eukaryotic cell are phosphorylated at some point, emphasizing the ubiquitous role of protein phosphorylation [29]. Phosphorylation is involved in many cellular processes such as metabolism, homeostasis, transcriptional and translational regulation, the degradation of proteins, cellular signaling and communication, proliferation, differentiation, and cell survival [28,29].

**Figure 1:**
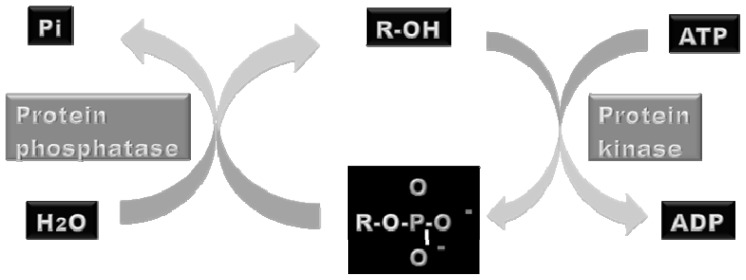
Illustration of the catalytical activities of protein kinases and phosphatases. Protein kinases catalyses the addition of a phosphoryl group to a target protein using adenosine triphosphate (ATP) or guanosine triphosphate (GTP) as phosphate donors. On the other hand, protein phosphatases catalyze the hydrolysis of the phosphopeptide bond. Orthophosphate (Pi) is produced by hydrolysis.

Protein phosphorylation is catalyzed by protein kinases using adenosine triphosphate (ATP) or guanosine triphosphate (GTP) as phosphate donors. The terminal γ phosphoryl group of ATP or GTP is transferred to the alcohol group of tyrosine by tyrosine kinases or of serine or threonine by serine/threonine kinases. Only those proteins in the intracellular environment, where ATP is abundant, are regulated by reversible phosphorylation, whereas proteins located in the extracellular matrix are not [30]. The phosphoryl groups are removed by protein phosphatases, which catalyze the hydrolysis of the phosphate bond under the production of orthophosphate (Figure 1).

Phosphorylation affects proteins structurally, thermodynamically and kinetically. The addition of a phosphoryl group brings two negative charges to the protein, which may change the catalytical activity and substrate binding efficiency of the protein. Phosphorylation is often involved in reaction cascades where a signal is amplified throughout the cell.

### 1.3. Phosphoproteomics: Useful in Signaling Pathways Research Studies

Phosphorylation is a reversible PTM, which plays a crucial role in the regulation of signaling pathways. It controls many biological responses such as cell growth, differentiation, invasion, metastasis and apoptosis. Current phosphoproteomics approaches are a powerful tool for monitoring global molecular responses to the activation or deactivation of signal transduction pathways. Many advances in mass spectrometry (MS) have enabled the identification and quantitation of thousands of known and novel phosphorylation sites [31,32]. Therefore, today’s scientists take heed of those strategies for functional phosphoprofiling studies of signaling pathways, for drug discovery and for the understanding of different diseases.

Abnormal phosphorylation is recognized as a cause or a consequence of many human pathologies or diseases, like cancer [33–36]. The deregulation of protein kinase functions is implicated at the onset of tumor formation and cancer progression. Therefore, kinase signaling pathways are a current major focus of biomedical research [37–39] in the development of therapeutic targets [40–42]. The activation state of phosphorylation-dependent signal transduction pathways in relation to the onset of diseases may result in the identification of novel drug targets or in the identification of novel disease markers. In the past, many gene expression profiling studies have been performed in the screening and evaluation of specific diseases related to the alteration of the gene expression [42].

Phosphoproteomics provides additional information, as it also offers a way in which to qualify and quantify the state of kinase-dependent pathways and provides detailed post-translational phosphorylation information [43,44]. The cellular proteome consists of many different proteins of which the phosphoproteins represent one-third, so it is difficult to identify all phosphoproteins from such complex biological samples. Thus, one of the main challenges for the applications of phosphoproteomics consists of successfully subtracting phosphoproteins from the whole cell lysates or tissues with the focus on identifying the low abundant phosphoproteins. This implies difficult and tedious processes, in which difficulty is increased due to the fact that phosphorylation of a protein is a reversible and highly dynamic event, which often takes place at multiple residues.

In order to understand the onset of certain cell biological events, one needs to understand the crosstalk between kinases, phosphatases and phosphoproteins in different signal pathways. For this, it is important to follow kinetic changes in protein phosphorylation at various time points after, for example, stimulation or stress of cells. Since it is somewhat laborious and difficult to obtain a global kinetic profile of protein phosphorylation on a broad scale, today, scientists chiefly tend to use liquid chromatography (LC) online MS techniques, in order to achieve high sensitivity analysis of phosphoproteomes with a high dynamic range. Moreover, it is crucial to couple complementary methods for separation and sequencing of phosphopeptides.

Large scale phosphoproteomics strategies [45–47] typically include enrichments of phosphopeptides using immobilized metal affinity chromatography (IMAC) or titanium dioxide (TiO_2_) resin, [48] followed by reverse phase LC coupled to electrospray ionization (ESI) tandem MS. Indeed, strong cation exchange coupled to IMAC permit phosphoproteome/large scale studies and novel phosphoenrichment methods (calcium phosphate [49] and secuencial elution from IMAC [50]) are being successfully used in phosphoproteome analysis from complex samples. On the other hand, the combination of collision induced dissociation (CID) and electron transfer dissociation (ETD)/electron capture dissociation (ECD) fragmentation is a very promising approach for phosphoproteomic studies [51,52] and also with the application of many of these technologies new insights into functional phosphorylation network cascades events can be discovered [52].

In addition, to simplify the phosphopeptides analysis by MS, it is a prerequisite to load them into reverse phase chromatographies. Those chromatographies can be packed by different materials: R3, C18 and/or graphite. Poros R3, disks C18, and powder graphite are materials containing long hydrocarbon chains, proven effective for the desalting and cleaning of ultra hydrophilic peptides, including phosphopeptides [53].

**Figure 2:**
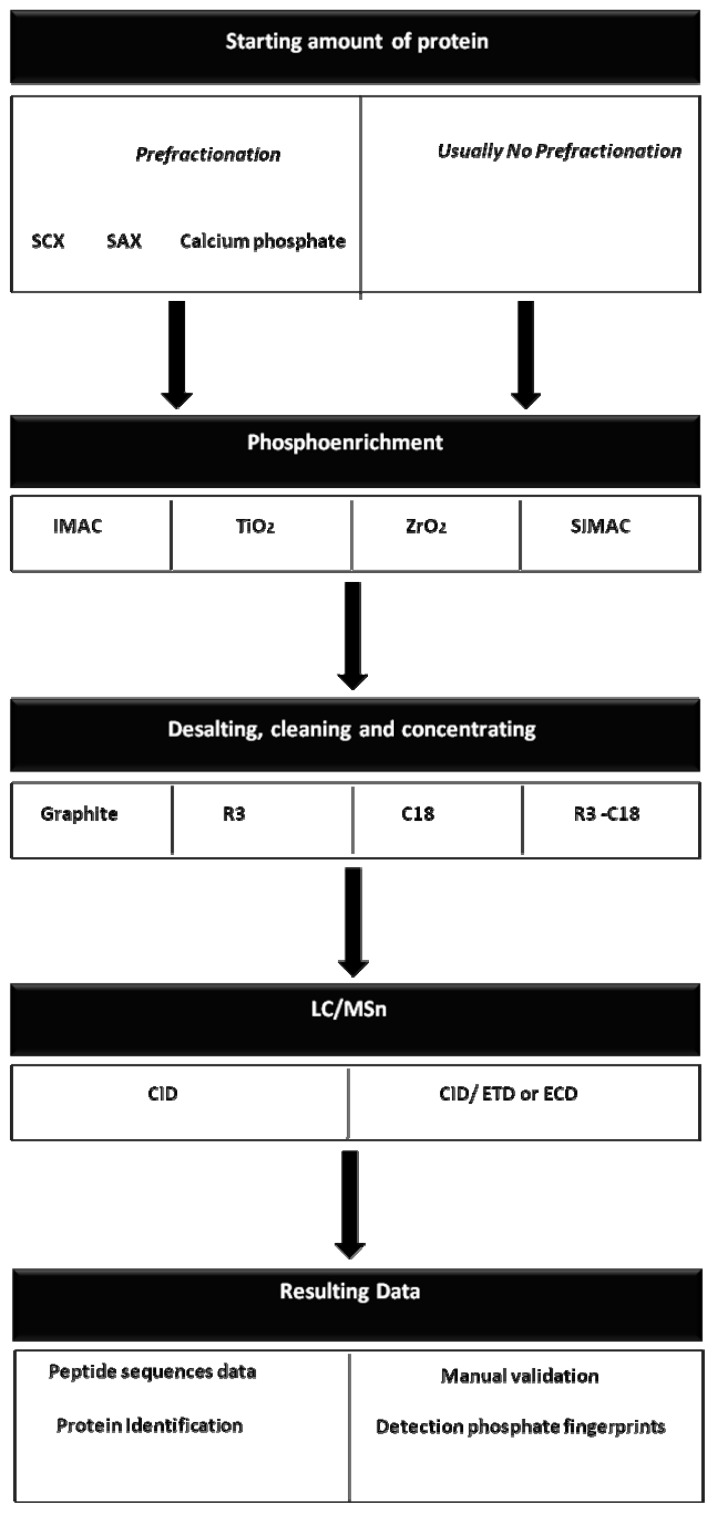
The workflow of current strategies for large-scale phosphoproteomics analyses. Different strategy-combinations can be coupled in order to recover and identify the maximum number of phosphopeptides.

In 1999, Gobom and co-workers [54] introduced a micro-column purification method in which a chromatographic resin was packed in the tip of a small, constricted GELoader tip, creating a micro-column. It is clear that various and different strategies have been established for functional phosphoproteomic clinical analyses. The flowthrough of current strategies for large scale phosphoproteomic analysis can be observed in Figure 2, while a summary of the different phosphoenrichment method properties can be observed in Tables 2 and 3.

We now aim to detail important concepts of specific and previously mentioned phosphoenrichment methods as a take home message. They can also be used as a guideline. The most common techniques to enrich for individual and/or global phosphorylation are IMAC and TiO_2_ [55], which are based on the high affinity of phosphate groups for metal ions such as Fe^3+^, Zn^2+^, Cu^2+^ and Ga^3+^. The negatively charged phosphopeptides will bind the positively charged metal ions by electrostatic interactions.

One of the main limitations associated with both phosphopeptide enrichments is the non-specific retention of non-phosphorylated acidic peptides, due to the weak affinity between negatively charged carboxylate and positively charged metal ions. However, conversion of carboxylate groups to esters effectively eliminates non-specific retention of non-phosphorylated peptides, although this has the drawback of increasing complexity in the subsequent MS analysis.

During the last seven years, TiO_2_ has emerged as the most common of the metal oxide affinity chromatography (MOAC) based phosphopeptide enrichment methods. This technique offers increased capacity compared to IMAC resins in order to bind and elute mono-phosphorylated peptides. TiO_2_ exploits the same principle as IMAC, and, is similarly prone to nonspecific retention of acidic nonphosphorylated peptides. However, when loading peptides in 2,5-dihydroxybenzoic acid (DHB) [56], glycolic and phthalic acids, nonspecific binding to TiO_2_ is reduced, thereby improving phosphopeptide enrichment without chemical modification of the sample. In addition, TiO_2_ is often considered to be interchangeable with IMAC. It works in similar levels of sample amounts (e.g., micrograms of protein) for the identification of phospho-sites by MS analysis.

On the other hand, SIMAC [50,57,58] appeared as a phosphopeptide enrichment tool, which exploits the properties of IMAC coupled to TiO_2_, making it possible to carry out more refined studies. Another phosphopeptide enrichment prior to mass spectrometric analysis is ZrO_2_ [58] and its principle is based on metal affinity chromatography like IMAC and TiO_2_. ZrO_2_ allows isolation of single phosphorylated peptides more selectively than TiO_2_ when using casein as protein models. It has, in fact, been successfully used in large-scale characterization of phosphoproteins.

Furthermore, a strategy that consists of fractionating and subsequently enriching phosphopeptides on a proteome wide scale is based on strong cation/anion exchange (SCX and SAX) chromatography. The principle of SCX/SAX phosphopeptide enrichment is based on the negative charged phosphate group (PO_3_^−^) of the phosphopeptides.

In cation exchange chromatography, a positively charged analyte is attracted to a negatively charged solid-support, whereas in anion exchange chromatography negatively charged molecules are attracted to a positively charged solid-support. SAX has previously been successfully combined with IMAC [59] and has resulted in greater recovery and identification by MS of mono-phosphorylated peptides originating from membrane proteins. SCX has, in a similar way, been combined with IMAC (Fe^3+^) and MS analysis, allowing the identification of thousands of phosphorylated residues from complex biological samples [59].

Calcium phosphate precipitation is a strategy that provides a useful pre-fractionation step to simplify and enrich phosphopeptides from complex samples. Zhang and co workers [49,60] have demonstrated that phosphopeptide precipitation by calcium phosphate combined with a two-step IMAC (Fe^3+^) procedure resulted in the observation of an increased number of phosphopeptides. This method consists of precipitating phosphopeptides by adding 0.5 M NaHPO_4_ and 2 M NH_3_OH to the peptide-mixture followed by 2 M CaCl_2_. The sample is vortexed and centrifuged, and subsequently the supernatant is removed before washing the pellet with 80 mM CaCl_2_. The washed pellet is dissolved in 5% of formic acid and the resulting peptide mixture is desalted by reversed phase chromatography before isolating the phosphopeptides by IMAC (Fe^3+^).

It is important to remark that titanium and zirconium were successfully applied to enrich phosphopeptides with the aid of aliphatic hydroxy acids such as lactic acid and beta-hydroxypropanoic acid, in order to reduce the interaction between acidic non-phosphopeptides and the metal oxides. Ishihama and co-workers [61] developed a novel method for phosphopeptide enrichment using aliphatic hydroxy acid-modified metal oxide chromatography (MOC).

This method removed the vast majority of non-phosphopeptides from phosphoprotein standard digests and large numbers of phosphopeptides could be readily identified. Ishihama and co-workers [61] coupled their methods with nano-LC-MS/MS systems and recovery of phosphopeptides in MOC varied greatly from peptide to peptide, ranging from a few percent to 100%, the average being almost 50%. Repeatability and linearity were satisfactory. In an examination of the cytoplasmic fraction of HeLa cells, more than 1000 phosphopeptides were identified using lactic acid-modified titania MOC and beta-hydroxypropanoic acid-modified zirconia MOC, respectively.

The overlap between phosphopeptides enriched by these two methods was 40%, and the combined results provided 1646 unique phosphopeptides. Custom-made MOC tips were prepared by Ishihama and co-workers [61] using C_8_ StageTips and metal oxide bulk beads (3 mg of beads/200-μL pipette tip). Prior to loading samples, the MOC tips were equilibrated with 0.1% TFA, 80% acetonitrile with hydroxy acids as selectivity enhancers (solution A). As the enhancers, glycolic acid, lactic acid, malic acid, tartaric acid and DHB were used at a concentration of 300 mg/mL and HPA was used at 100 mg/mL. The digested standard phosphoprotein mixture (15 μL) was mixed with 100 μL of solution A and loaded on the MOC tip. After successive washing with solution A and solution B (0.1% TFA, 80% acetonitrile), 0.5% ammonium hydroxide was used for elution. They developed highly selective enrichment methods for phosphopeptides using lactic acid-modified titanium and HPA-modified zirconium MOC tips. Both methods were applied to the cytoplasmic fraction of HeLa cells and over a thousand phosphopeptides were identified. The combination of these methods will, no doubt, be useful for proteome-wide experiments to study cell signaling networks, which require the enrichment of many phosphopeptides from real complex mixtures with a wide dynamic range [61].

Finally, since most phosphopeptide analysis is nowadays performed by MS and this technique is sensitive to contaminants such as salts, it is necessary to clean the samples prior to analysis, generally by reversed phase chromatography combining POROs R3 with C18 Disks and also graphite powder [53,62–64]. Poros R3, C18 Disks and graphite powder are materials containing long hydrocarbon chains, proven to be effective for the desalting and cleaning of very hydrophilic peptides, including phosphopeptides [56,65].

In 1999, Gobom and co-workers introduced a micro column purification method in which a chromatographic resin was packed in the tip of a small, constricted GELoader tip, creating a micro-column [62]. Using a chromatographic approach, GELoader tips packed with R3, C18 or graphite material, contaminants like salts can be separated from the phosphopeptides. In fact, by using RP chromatography, molecules such as proteins, peptides and nucleic acids are separated according to their hydrophobicity. In addition to the removal of salts, these techniques also facilitate a concentration of the sample by the use of a low elution volume. This further improves the sensitivity and quality of the subsequent mass spectrometric analysis.

Zou and co-workers [66] have conducted comprehensive clinical phosphoproteomics liver research studies to date. This work contributes to the understanding of phosphorylation mechanisms at the systemic level and provides powerful methodology for the general analysis of *in vivo* PTMs regulating sub-proteomes. They presented a novel software package of iGPS for the prediction of *in vivo* site-specific kinase-substrate relations from the phosphoproteomic data. They modelled protein phosphorylation networks and observed that the eukaryotic phospho-regulation is poorly conserved at the site and substrate levels. They conducted a large-scale phosphorylation analysis of human liver and experimentally identified 9719 p-sites in 2998 proteins. Using iGPS, they were able to predict 12,819 potential ssKSRs among 350 PKs and 962 substrates for 2633 p-sites in a human liver sample. They provided the largest data set of the human liver phosphoproteome together with computational analyses that can be useful for further experimental consideration. The phosphopeptides were enriched by the digestion of human liver lysate by Ti^4+^-IMAC Microspheres [67]. Peptide mixtures which were first incubated with the Ti^4+^-IMAC microsphere suspension (10 mg/mL in 80% ACN, 0.1% TFA) for 30 min, then were washed with a solution containing 50% ACN, 6% TFA and 200 mM NaCl, followed by washing with 30% ACN/0.1% TFA. Finally, the enriched phosphopeptides were eluted with 10% NH_3_·H_2_O and dried by vacuum centrifugation [67].

### 1.4. Clues to Sample Preparation for Efficient Clinical Phosphoproteomic Studies

Sample preparation and treatment steps impact on all the later assayed steps and it is hence critical for unequivocal identification, confirmation and quantification analysis. Moreover, a poorly treated sample may invalidate the whole assay. Sample preparation is, in most cases, especially in complex samples, meant to be the isolation and/or concentration of some components of interest, making the compounds more suitable for the separation and the detection steps. All the treatments of the sample of interest must later facilitate the structure elucidation.

In covering phosphoproteome maps, it is crucial to couple different methods and if sample preparation, treatments and instrumentation steps are optimized specifically for the sample of interest, thousands of phosphopeptides may be identified. It has recently been demonstrated that the yield on recovering phosphopeptides is more dependent on the presence of salts and detergents when using IMAC (Fe^3+^) compared to TiO_2_ [55,68]. Indeed, this is also true when applying the lyophilization procedure to the sample of interest [69].

Most of the complex samples need to be treated with salts, different detergents (e.g., triton, chaps) or also reagents such as urea and dithiothreitol in order to extract the proteins. When performing sample treatments containing salts or detergents, it is important to notice that the presence of those reagents does not affect the binding capacity of the phosphopeptides from the sample to the metal ions from the metal affinity chromatography. A possible solution for avoiding this problem could be the precipitation of the complex sample proteins using acetone at −20 °C for 2 h, therefore the pellet containing the proteins of interest would be free from salts and detergents. The proteins as a pellet can then be re-dissolved in the necessary buffers (NH_4_HCO_3_) in order to correctly carry out tryptic digestions and subsequently be loaded onto phosphoenrichment microtips. On the other hand, when performing phosphoenrichment methods followed by desalting and cleaning reverse phase chromatography in order to analyze the sample by LC, it is necessary to reduce the volume of the solution containing phosphopeptides. We observed that when completely drying this solution, the identified phosphopeptides were dramatically reduced, which has concordance with Corthals and co-workers [70]. This can be solved by partial reduction of the volume containing the phosphopeptides. Moreover, all these critical steps must be optimized for the characteristics of the sample of interest, especially for complex samples, as it was demonstrated just for certain simple samples (standards such as ovoalbumin or casein) the complete lyophilization of the phosphopeptides and some specific detergents do not decrease the number of phosphopeptides recovered.

On the other hand, the material quantity to be packed in the metal affinity and reverse phase chromatographies, and the sample amount to be analyzed, must be adjusted to efficiently bind, elute, and desalt phosphopeptides according to the different sized tips used (GelLoader tip, p10, and p200). This step also requires some prior tests and optimizations with the sample of interest.

Another important and well-established issue is the addition of phosphatase inhibitors to avoid losing phosphopeptides [71,72]. Some time ago, the efficiency of adding phosphatase inhibitors to the sample in order to avoid losing phosphopeptides, was demonstrated. This is due to the fact that phosphorylation is a labile and reversible PTM; indeed it is blocked in a biological way by the very same phosphatases in cell systems. In addition, phosphatases coming from outside the sample (artifacts) can easily degrade the phosphate groups. This is a well established, but really important step, for the sample preparation. Some excellent reviews on this topic have appeared and have summarized the fundamental aspects of sample extraction [73,74], which were necessary with special considerations of phospho-time studies (Table 4).

### 1.5. MS and Phosphorylation Analysis of Proteins

The development of techniques during the 1980s and 1990s that allowed for the ionization of proteins and peptides began to make mass spectrometers extremely valuable to biochemists. The two most notable techniques developed were matrix-assisted laser desorption/ionization (MALDI) [75] and ESI [76], for which the primary developers of these two techniques were awarded the 2002 Nobel Prize for chemistry. Today, both MALDI and ESI are coupled with numerous types of mass analyzers, including ion trap, time-of-flight (TOF), and Fourier transform ion cyclotron resonance to create mass spectrometers that are sold by a variety of analytical instrumentation companies. Indeed both types of ionization (MALDI and ESI) allow phosphoproteomics studies to be carried out but with very different outcomes. The MALDI ionization mechanism often leads to the suppression of phosphorylated peptides and it is much more difficult to fragment and sequence peptides generated with MALDI.

The neutral loss of phosphoric acid (H_3_PO_4_) is a classical signature that is used to identify phosphopeptides by MALDI-TOF-MS and phosphorylation sites by ESI-MS/MS coupled to LC. Even when starting from samples that are enriched in phosphoproteins or phosphopeptides, this neutral loss (NL) of phosphoric acid might lead to an ambiguous identification of phosphorylation sites from MS/MS data. Due to this, phosphate fingerprints are a necessary manual evaluation tool in MS, to validate the phosphopeptides identified by MS. Firstly, the phosphate group is removed from serine and threonine residues by gas phase β-elimination as phosphoric acid (H_3_PO_4_) due to CID or metastable fragmentation [77]. This gives rise to the loss of 98 Da from the phosphorylated residue (phosphate group 80 Da and water 18 Da), which is used as a positive identification of phosphoserine and phosphothreonine. Secondly, if the phosphate group is positioned on a serine residue, a peak 69 Da below the peak originating from the β-eliminated phosphopeptide will appear. Therefore, this peak appears due to the conversion of phosphoserine into dehydroalanine during β-elimination. The presence of dehydroalanine indicated by the differences of 69 Da and 167 Da (98 + 69 Da) are indicators of a phosphoserine in the peptide sequence. On the other hand, the tyrosine immonium ion of 136 Da is commonly observed in the spectrum of a peptide containing tyrosine, and a phosphorylated tyrosine residue in the sequence results in an immonium ion at 216 Da (136 + 80 Da), which is a diagnostic ion for a phosphotyrosine [78].

Indeed, during CID fragmentation, phosphopeptides are prone to partial or complete loss of phosphoric acid, while minimal fragmentation takes place along the peptide backbone, revealing little or no information about the peptide’s sequence. The recently developed fragmentation techniques of ECD and ETD have been shown to fragment the peptide backbone while leaving the phosphoserine/phosphothreonine intact. Not only have ECD and ETD been used to sequence phosphopeptides, ECD has also been used to sequence phosphoproteins [79]. While both fragmentation techniques (ECD and ETD) are of great utility to fragmenting phosphopeptides, to date ECD has been exclusively coupled to Fourier transform ion cyclotron resonance instruments, the most expensive type of MS instrumentation available. ETD, on the other hand, is likely to be very useful since it is easily adapted to more cost efficient ion trap mass spectrometers that are commonly used for protein and peptide analysis. At the present moment, both ETD and ECD techniques are more widely available to make a broad impact in characterizing phosphoproteins. Current mass spectrometers combining CID and ETD fragmentation allow for such characteristics as high mass resolution, high mass accuracy, sensitivity, and dynamic concentration range in phosphorylation studies. As it is difficult to discuss the advantages and limitations of each type of instrument within the broad field of qualitative and quantitative phosphoprotein analysis, and since the development of mass spectrometers is occurring at a rapid pace, we suggest that experienced biological MS labs be consulted during the planning stages of phosphoprotein experiments to determine the most appropriate instrumentation and methodologies likely to provide the desired results.

Furthermore, two general MS different strategies have been proposed and are being used in order to improve confidence in these phosphorylated identifications. Firstly, additional fragmentation of the remaining precursor (MS3) has been successfully used to map the phosphorylation sites. Secondly, by combining the selection of the phosphorylated parent ion in MS with selection of the dephosphorylated ion or associated product in MS/MS, this approach introduces high specificity, allowing the characterization of low abundance phosphorylation sites [80].

Although these two previously mentioned methods have permitted the identification of thousands of phosphorylated sites, phosphoproteome analysis carried out in an automated mode (MS2, MS3) can generate some ambiguous data. In such an endeavor, today mass spectrometrists tend to use the multiple reactions monitoring, especially to identify low levels of phosphorylated proteins. This last third method consists of taking advantage of the protein information available, which can be used to predict precursor and fragment ion values in order to trigger dependent ion scans on a hybrid quadrupole linear ion trap instrument. Indeed, multiple reactions monitoring was used by Whyte and co-workers [81] achieving an extremely high yield in identifying tyrosine phosphorylated kinases. This technology is already available and applicable for studying signaling networks with relevant potential use in pharmaceutical research for the design of new therapeutic drugs. It is important to point out that whereas shotgun proteomics aims at sequencing all peptides that survived the purification method, multiple reaction monitoring aims at detecting and sequencing only desired peptides, either based on a particular sequence or based on a particular reaction they undergo in the collision zone—like the loss of a phosphoric acid. Multiple reaction monitoring scans generate filtered mass spectra, filtered for specific reactions the peptides undergo in the collision zone.

### 1.6. Validation of Phosphorylated Peptides/Proteins

One concern which the trend toward massive phosphoproteomics datasets is the inability to properly validate MS/MS spectral assignments for each phosphorylated peptide. Performed properly, spectral validation requires the assignment of all abundant ions in the spectrum necessitating a significant time commitment. To bypass this bottleneck, many MS labs have utilized decoy search strategies and statistical methods to estimate false positive identification rates. Statistical validation is a greater concern for phosphoproteomics in comparison to the larger field of proteomics since each phosphorylation site is typically defined by a single MS/MS spectrum. Unfortunately, it is impossible to tell if any given phosphorylation site is correctly identified using statistical methods, as the MS/MS spectrum remains invalidated.

False positive identifications are particularly dangerous for biologists interested in studying the function of these selected phosphorylation sites, as each phosphorylation site may take around 2 years to be fully investigated. This situation is exacerbated because most biologists do not have the requisite expertise to assess the accuracy of the assignment even if the raw MS/MS spectrum is provided and it is often mistakenly assumed that all published phosphorylation assignments are correct. On the other hand, manual validation of the phosphorylated peptides/proteins is a good challenge, but it implies high expertise with reading the spectra [82].

In a simple manner, we summarize and detail the manual validation of the phospho-data (assignments of the phosphate group on specific amino acids) obtained in a MS experiment during CID operations. When peptide ions are fragmented via CID, series of y- and b-ions are formed [83,84]. The peptide sequence is obtained by correlating mass difference between peaks in the y-ion series or between peaks in the b-ion series with amino acid residue masses. The CID fragmentation occurs mainly on the peptide backbone and sequence information is obtained. Related to phosphotyrosine residues, partial neutral loss is observed (HPO_3_, 80 *m/z*) in MS2 mode, and the phosphate group on tyrosine (tyr) residues is more stable than on serine (ser) and threonine (thr) residues. The phospho-finger-print characteristic of phosphotyrosine is the phosphotyrosine immonium ion (~216 Da) [83].

Via the MS3 mode, the ion originating from NL of phosphoric acid (H_3_PO_4_) can be selected for further fragmentation. Then the selected ion is automatically selected for further fragmentation after NL fragmentation. Therefore it is possible to add extra energy for the fragmentation of peptide backbone. Nevertheless, the MS3 mode requires that the phosphorylation on ser and thr residues are labile and conventional fragmentation via CID commonly results in the partial NL of H_3_PO_4_, (98 *m/z*) in MS2 mode. This is due to the gas phase β-elimination of the phosphor-ester bond, thus dehydroalanine (ser ~69 Da) and dehydro-2-aminobutyric acid (thr ~83 Da) are generated [84].

### 1.7. Quantitative MS Methods that Rely on Stable Isotope Incorporation for Phosphorylated Peptides/Proteins Measurements

Proteins containing amino acids with one or more of the stable isotopes of ^2^H, ^13^C, ^15^N or ^18^O can be used as internal standards by the addition, at an early stage of the analysis, of a complex protein sample. There are two approaches for introducing a stable isotope into proteins or peptides: (a) metabolic labeling using whole cells grown in culture (e.g., SILAC—Stable Isotope Labelling with Amino acid in cell Culture) or (b) chemical labeling (e.g., iTRAQ—Isobaric Tag for Relative and Absolute Quantitation, ICAT—Isotope-Coded Affinity Tags).

Measuring the changes in phosphorylation is critical for understanding the biology of a phosphorylation event, since protein phosphorylation is very dynamic and in constant change throughout the life of a cell. We restrict the discussion here to three MS-based quantitation strategies which have direct utility towards measuring changes in protein phosphorylation: SILAC, iTRAQ, and AQUA. Other chemical labeling techniques that rely on stable isotope incorporation using e.g., ^18^O labeled water during trypsin digestions and stable isotope incorporation ICAT can also be considered with relevant information, but will not be described here.

#### 1.7.1. SILAC

Stable isotope labeling by amino acids in cell culture (SILAC) is a quantitative method based on *in vivo* labeling of proteins in cell cultures with amino acids that contain stable isotopes (non radioactive, e.g., ^2^H, ^13^C and ^15^N) [85,86]. In its simplest form, two separated cell cultures are grown in a pair-wise fashion; for example, culture A might be yeast cells grown under “normal” conditions (light conditions) while culture B might be yeast cells grown in the presence of a stress condition. The growth conditions of the cells are identical (except for the presence of the stress-stimuli), but the growth media of culture B has an essential amino acid (one not synthesized by the cell) replaced with an isotopically “heavy” form of that amino acid (e.g., ^13^C_6_-arginine).

A number of cell lines have been used for SILAC experiments and the growth and morphology of the cells has not been affected by the isotopically labeled amino acid [59,86,87]. After approximately five rounds of doubling, cellular proteins are essentially 100% labeled with the selected amino acid. After culturing, the light and heavy cell populations are combined (1:1) into one pool and the proteins are isolated. The protein pool is then digested with a protease, typically trypsin, to form a peptide pool that is analyzed by MS. Each peptide analyzed will be present in two forms: the light and the heavy form. The two forms have the same chemical properties, so they have approximately the same chromatographic retentions, ionization efficiencies, and fragmentation characteristics, but they are distinguishable based on the mass difference due to the heavy isotope incorporation in the selected amino acid. The peak signals produced by the light and heavy forms of a peptide are measured by the mass spectrometer, and a relative quantification of that peptide from the two cultures is calculated.

Tandem MS is also performed in the same experiment in either the light or the heavy form. Therefore, the identity of the peptide and the protein is determined. In fact, all peptides, both phosphorylated and non-phosphorylated, containing the isotopically labeled amino acid, are available for relative quantification by SILAC. The SILAC method is compatible with the previously mentioned enrichment of phosphoproteins/phosphopeptides including the immunoprecipitation of a target protein [88]. To assist the enrichment of phosphopeptides in the SILAC method, the combination of SCX chromatography and IMAC, have been employed after proteolytic digestion [59]. This approach enriches the phosphopeptides and helps to remove non-phosphorylated peptides that can act as disturbance in the quantification experiment.

#### 1.7.2. iTRAQ

The second method for the global quantification of proteins and protein modifications is an *in vitro* chemical labelling procedure called iTRAQ. The iTRAQ reagent consists of two to eight isobaric (same nominal mass) tags that can be used to label two to eight separate protein samples; for example, one sample might be “normal” yeast cells while the three remaining samples might be yeast cells grown at three different concentrations of stress treatment.

The iTRAQ tags contain three regions: a peptide reactive region, a reporter region, and a balance region [89]. The peptide reactive region of the tag consists of an NHS ester and is designed to react with the *N*-termini and lysines of peptides after protease digestions. In the case of 4-plex iTRAQ, the four reporter groups appear in the tandem mass spectrum at *m*/*z* 114, 115, 116, and 117. The attached balance groups are designed to make the total mass of the balance and reporter group 145 Da for each tag, resulting in balance groups of 31 Da, 30 Da, 29 Da, and 28 Da, respectively.

Protein samples quantification are separately isolated and digested proteolytically and each sample is chemically labeled with one of the iTRAQ reagents. After labeling, the samples are combined and subsequently analyzed by MS. As the iTRAQ reagents are isobaric, identical peptides from each sample will have identical masses, therefore there is no division of the precursor signals in the first stage of mass analysis that could lead to increased spectral complexity by the combination of multiple samples. Additionally, the isobaric nature of the reagent increases the ion population for a given peptide by summarizing the amount of a peptide from each sample. This makes the detection of the peptides more sensitive.

During tandem MS, fragmentation takes place along the peptide backbone and also between the reporter and balance region of the tag which facilitates the quantification based on the intensity of the reporter ions. The reporter ions in the tandem mass spectrum are, in general, more intense than the fragment ions. The relative amounts of these reporter ions correspond to the relative amounts of the peptides present in the four samples. In contrast to SILAC and AQUA (described below), it is during tandem MS experiments, and not during the first stage of mass analysis, that relative quantification of peptides takes place.

Phosphoproteins can be analyzed in an identical manner as well as non-phosphorylated proteins with iTRAQ. The iTRAQ reagent labels phosphopeptides to the same degree as non-phosphorylated peptides and it does not affect the stability of phosphopeptides. Enrichment strategies, such as IMAC [72,90,91] or immunoprecipitation with anti-phosphotyrosine antibodies [91], have been utilized to remove non-phosphorylated peptides to focus the analysis on site-specific phosphorylation. Also, since iTRAQ is an *in vitro* labeling procedure, it can be applied to clinical samples such as tumour tissues and fluids (e.g., serum, urine, blood). iTRAQ has been described as a very powerful method for the quantification of phosphorylation on a proteomic scale. In addition, White and colleagues [92] applied iTRAQ combined with MRM for phospho quantitative analysis of signaling networks, identifying and quantifying 222 tyrosine phosphorylated peptides, obtaining an extremely high percentage of signaling nodes covered.

#### 1.7.3. AQUA

The AQUA strategy provides an absolute quantification of a protein of interest [93] In the AQUA method, a peptide from the protein of interest is constructed synthetically containing stable isotopes, and the isotopically labeled synthetic peptide is called AQUA peptide. The synthetic peptides can be synthesized with modifications such as phosphorylation to allow for the direct, quantitative analysis of post-translationally modified proteins.

The stable isotopes are incorporated into the AQUA peptide by using isotopically “heavy” amino acids during the synthesis process of the peptide (native peptide) of interest. Therefore, the synthetic peptide has a mass increase of e.g., 10 Da, due to the incorporation of a ^13^C_6_ and ^15^N_4_-arginine into the synthetic peptide, compared to the native peptide. Although the mass difference between the native and the synthetic peptide allows the mass spectrometer to differentiate between the two forms, both forms have the same chemical properties, resulting in the same chromatographic retention, ionization efficiency, and fragmentation distribution.

In AQUA experiments, a known amount of the isotopically labeled peptide is added to a protein mixture, which is proteolytically digested and later analyzed by MS. Since the native peptide and its synthetic counterpart have the same chemical properties, the MS signal from the quantified synthetic peptide can be compared to the signal of the native peptide. This finally allows the absolute quantification of the peptide to be determined [94]. Multiple AQUA peptides can be used to quantify multiple proteins in a single experiment.

## 2. Phosphoproteomic Technologies and Platforms

There are a number of developments in the phosphopeptide enrichment, e.g., Kettenbach and Gerber [95] have demonstrated a straightforward and reproducible approach for the broad scale analysis of protein phosphorylation that relies on a single phosphopeptide enrichment step using titanium dioxide microspheres from whole cell lysate digests and compared it to the well-established strong cation exchange-TiO_2_ workflow for phosphopeptide purification on a proteome-wide scale. They have established and validated a robust approach for proteome-wide phosphorylation analysis in a variety of scenarios that is easy to implement in biomedical research and translational settings.

On the other hand, the ability of high-field asymmetric waveform ion mobility spectrometry (FAIMS) to separate multiply charged peptide ions from chemical interferences confers a unique advantage in phosphoproteomics by enhancing the detection of low abundance phosphopeptides. LC-FAIMS-MS experiments performed on TiO_2_-enriched tryptic digests from *Drosophila melanogaster* provided a 50% increase in phosphopeptide identification compared to conventional LC-MS analysis. FAIMS can also be used to select different population of multiply charged phosphopeptide ions prior to their activation with either collision activated dissociation (CAD) or ETD. Importantly, FAIMS enables the resolution of coeluting phosphoisomers of different abundances to facilitate their unambiguous identification using conventional database search engines [96].

Masuda *et al*. [97] have developed a miniaturized LC-MS system with a high-recovery phosphopeptide enrichment protocol that allows phosphoproteome analysis of 10^4^ cells. In the enrichment protocol, the key step is to add sodium deoxycholate and sodium lauroyl sarcosinate to buffer solution for protein extraction and digestion and to omit any subsequent desalt/desurfactant step before phosphopeptide enrichment. The phosphopeptides enriched by hydroxy acid-modified metal oxide chromatography are directly injected onto a miniaturized LC column using a nitrogen-pressure-driven cell, instead of switching valve-type injectors. The miniaturized analytical column of 25 μm diameter provided a 3.6-fold improvement in sensitivity over the conventional 100 μm diameter column. This analytical system can provide approximately 80-fold improvement on average in the LC-MS response, and it can identify 1011 unique phosphorylated sites based on 995 unique phosphopeptides from a single analysis of 10^4^ HeLa cells (approximately 1 μg of proteins). This is the most sensitive phosphoproteomics system that has so far been reported for proteome-wide analysis of *in vivo* phosphorylation in mammalian cells.

By functional phosphoproteomics, Sudhir *et al*. [98] studied the molecular mechanics of oncogenic Ras signaling using a pathway-based approach. They identified Ras-regulated phosphorylation events using label-free comparative proteomics analysis of immortalized human bronchial epithelial cells with and without the expression of oncogenic Ras. Many proteins were newly identified as potential targets of the Ras signaling pathway. 60% of the Ras-targeted events consisted of a pSer/Thr-Pro motif, indicating the involvement of proline-directed kinases. By integrating the phosphorylated signatures into the Pathway Interaction Database, they further inferred Ras-regulated pathways (including MAPK signaling and other novel cascades) in governing diverse functions, such as gene expression, apoptosis, cell growth, and RNA processing. Their findings may aid to extend the understanding on Ras signaling in cancer.

The development of molecular tests for clinical use is constrained by the limited availability of fresh frozen samples. Gámez-Pozo *et al*. [99] have reported that phosphopeptide analysis in human archival formalin-fixed paraffin-embedded cancer samples based on IMAC followed by LC coupled with tandem MS and selected reaction monitoring techniques. They indicated the equivalence of detectable phosphorylation rates in archival formalin-fixed paraffin-embedded and fresh frozen tissues. This work paves the way for the application of shotgun and targeted phosphoproteomics approaches in clinically relevant studies using archival clinical samples.

Pierobon *et al*. [100] have worked out a patient-specific circuit diagram provides key information that identifies critical nodes within aberrantly activated signaling which may serve as drug targets for individualized or combinatorial therapy. The protein arrays provide a portrait of the activated signaling network by the quantitative analysis of the phosphorylated or activated state of cell signaling proteins. Based on the growing realization that each patient’s tumor is different at the molecular level, the ability to measure and profile the ongoing phosphoprotein biomarker repertoire provides a new opportunity to personalize therapy based on the patient-specific alterations.

Novel and improved computational tools are required to transform large-scale proteomics data into valuable information of biological relevance. Courcelles *et al*. [101] have developed the ProteoConnections, a bioinformatics platform tailored to address the pressing needs of proteomics analyses. They showed that combined proteomics and bioinformatics analyses revealed valuable biological insights on the regulation of phosphoprotein functions via the introduction of new binding sites on scaffold proteins or the modulation of protein-protein, protein-DNA, or protein-RNA interactions.

## 3. Phosphoproteomic Studies of Signaling Pathways and Kinase Inhibitors

CXCL12 (SDF-1) is a chemokine that binds to and signals through the seven transmembrane receptor CXCR4. The CXCL12/CXCR4 signaling axis has been implicated in both cancer metastases and HIV-1 infection. A more complete understanding of CXCL12/CXCR4 signaling pathways may support efforts to develop therapeutics for cancer. MS-based phosphoproteomics has emerged as an important tool in studying signaling networks in an unbiased fashion. Wojcechowskyj *et al.* [102] employed SILAC quantitative phosphoproteomics to examine the CXCL12/CXCR4 signaling axis in the human lymphoblastic CEM cell line. They quantified 4074 unique SILAC pairs from 1673 proteins and 89 phosphopeptides were deemed CXCL12-responsive in biological replicates. Several well-established CXCL12-responsive phosphosites were confirmed in their study, such as AKT (pS473) and ERK2 (pY204). They also validated two novel CXCL12-responsive phosphosites by Western blot, *i.e.*, stathmin (pS16) and AKT1S1 (pT246). Pathway analysis and comparisons with other phosphoproteomics datasets revealed that genes from CXCL12-responsive phosphosites are enriched for cellular pathways that have previously been linked to CXCL12/CXCR4 signaling, such as the T cell activation, epidermal growth factor, and mammalian target of rapamycin signaling pathways. Several of the novel CXCL12-responsive phosphoproteins provide an attractive list of potential targets for the development of cancer metastasis and HIV-1 therapeutics.

Indeed, aberrant signaling causes many diseases and manipulating signaling pathways with kinase inhibitors has emerged as a promising area of drug research. Most kinase inhibitors target the conserved ATP-binding pocket; therefore specificity is a key concern. Pan *et al*. [103] have introduced a complementary approach to evaluate the effects of kinase inhibitors on the entire cell signaling network. They used triple labeling stable isotope labeling by amino acids in cell culture (SILAC) to compare cellular phosphorylation levels for control, epidermal growth factor stimulus, and growth factor combined with kinase inhibitors. Of thousands of phosphopeptides, less than 10% had a response pattern indicative of targets of U0126 and SB202190, two widely used MAPK inhibitors. In contrast to MAPK inhibitors, dasatinib (a clinical drug directed against BCR-ABL which is the cause of chronic myelogenous leukemia) affected nearly 1000 phosphopeptides. In addition to the proximal effects on ABL and its immediate targets, dasatinib broadly affected the downstream MAPK pathways. This assay is streamlined and generic which may become a useful tool in kinase drug development.

Phosphoproteomics may enable monitoring of altered signaling pathways as a means of stratifying tumors and facilitating the discovery of new drugs. Using quantitative phosphoproteomics, Li *et al*. [104] have identified peptides corresponding to autophosphorylation sites of these tyrosine kinases that were inhibited in a concentration-dependent manner by dasatinib. Using drug-resistant gatekeeper mutants, SRC-family kinases (particularly SRC and FYN) and EGFR were shown to be relevant targets for dasatinib action. This provides a system-level view of dasatinib action in cancer cells, which suggests both functional targets and a rationale for combinatorial therapeutic strategies.

Actually, there is an urgent need to measure phosphorylated cell signaling proteins in cancer tissue for the individualization of molecular targeted kinase inhibitor therapy. However, phosphoproteins fluctuate rapidly following tissue procurement. It has been reported that a single paraffin block biomarker and histology preservative preserves the phosphorylation state of several signaling proteins at a level comparable to snap-freezing while maintaining the full diagnostic immunohistochemical and histomorphologic detail of formalin fixation. This new tissue fixative has the potential to greatly facilitate personalized medicine, biobanking, and phosphoproteomics research [105].

In the last decade, large-scale MS-based phosphoproteomics studies of receptor tyrosine kinases (RTKs) have generated a compendium of signaling networks that are activated downstream of these receptors. There are a number of important examples for the field to keep pace with new advances in RTK biology (e.g., EGFR, c-Met, and Flt3 receptors) which will greatly benefit from the power of phosphoproteomics, including: (a) validating oncogenic RTK mutants identified in cancer genome sequencing efforts; (b) spatial RTK signaling networks; and (c) understanding crosstalk and co-activation between members of the RTK superfamily [106].

## 4. Lung Cancer Phosphoproteomic Studies

In a recent study of Gámez-Pozo *et al*. [107], protein extracts were obtained from fresh frozen normal lung and non-small cell lung cancer (NSCLC) samples with phosphopeptide enrichment followed by LC-MS/MS. Subsequent label-free quantification and bioinformatics analyses including gene ontology and interactome analyses have identified signaling pathways altered on tumor tissue. Two proteins have been identified, PTRF/cavin-1 and MIF, which were differentially expressed between normal lung and NSCLC. These potential biomarkers were validated using Western blot and immunohistochemistry. The application of discovery-based proteomics analyses in clinical samples allowed the identification of new potential biomarkers and therapeutic targets in NSCLC.

The identification of key pathways dysregulated in NSCLC is an important step toward understanding lung pathogenesis and developing new therapeutic approaches. Reverse phase protein lysate arrays were used to compare signaling pathways between NSCLC tumors and paired normal lung tissue, as well as to assess their association with clinical outcome. A four-protein signature (p70S6K, cyclin B1, pSrc (Y527), and caveolin-1) was found to be an histological independent classifier for tumor *vs* normal with a predicted accuracy of 83%. In the tumors from patients with resected NSCLC, the expressions of proteins in the energy-sensing AMPK pathway (pLKB1, AMPK, p-Acetyl-CoA, and pTSC2), adhesion, EGFR, and Rb signaling pathways were revealed to be inversely associated with NSCLC recurrence. This data provides evidence for dysregulation of several pathways (including those involving energy sensing and adhesion) that are potentially associated with NSCLC pathogenesis and disease recurrence [108].

Patients with lung cancer often present with metastatic disease and therefore have a very poor prognosis. The recent discovery of several novel ROS RTK molecular alterations in NSCLC presents a therapeutic opportunity for the development of new, targeted treatment strategies. Jun *et al*. [109] recently reported that the NSCLC-derived fusion CD74-ROS, which accounted for 30% of all ROS fusion kinases in NSCLC, was an active and oncogenic tyrosine kinase. They found that CD74-ROS expressing cells were highly invasive *in vitro* and metastatic *in vivo*. Using quantitative phosphoproteomics, they uncovered a mechanism by which CD74-ROS activated a novel pathway driving cell invasion. Expression of CD74-ROS resulted in the phosphorylation of the extended synaptotagmin-like protein E-Syt1. They further demonstrated that the expression of CD74-ROS in non-invasive NSCLC cell lines readily conferred invasive properties that paralleled the acquisition of E-Syt1 phosphorylation. It is interesting to find that E-Syt1 is a mediator of cancer cell invasion and molecularly defined ROS fusion kinases may serve as therapeutic targets in the treatment of NSCLC.

Raf-1 kinase inhibitor protein (RKIP) has been reported to negatively regulate signal kinases of major survival pathways. RKIP activity is modulated in part by phosphorylation on serine 153 by protein kinase C, which leads to dissociation of RKIP from Raf-1. RKIP expression is low in many human cancers and it represents an indicator of poor prognosis and/or induction of metastasis. A study has examined the expression levels of both RKIP and phospho-RKIP in human lung cancer by tissue microarray proteomics technology. It was found that the phospho-RKIP levels slightly decreased in metastatic lesions. The normal expression levels of phospho-RKIP, in contrast to total RKIP, displayed significant predictive power for predicting a more favorable survival compared to lower levels [110].

## 5. Conclusions and Future Perspectives

Cells are highly responsive to their environment. Regulation of protein phosphorylation is a reversible modification plays an important role in many cellular processes, particularly in signal transduction. Protein phosphorylation affects most eukaryotic cellular processes and its deregulation is considered a hallmark of cancer and other diseases. With the completion of the human genome sequence, biomedical sciences have entered the omics era, principally due to high-throughput genomics techniques and the application of MS. Recent developments in phosphoprotein/phosphopeptide enrichment strategies and quantitative MS have resulted in robust pipelines for high-throughput characterization of phosphorylation in a global fashion [111]. Today, it is possible to profile site-specific phosphorylation events on thousands of proteins in a single experiment. The potential of this approach is already being realized to characterize signaling pathways that govern oncogenesis. In addition, chemical proteomic strategies have been used to unravel targets of kinase inhibitors, which are otherwise difficult to characterize [112].

The availability of enrichment methods combined with sensitive MS instrumentation has played a crucial role in uncovering the dynamic changes and the large expanding repertoire of this reversible modification. The structural changes imparted by the phosphorylation of specific residues afford exquisite mechanisms for the regulation of protein functions by modulating new binding sites on scaffold proteins or by abrogating protein-protein interactions. However, the dynamic interplay of protein phosphorylation does not occur randomly within the cell but is rather finely orchestrated by specific kinases and phosphatases that are unevenly distributed across subcellular compartments. This spatial separation not only regulates protein phosphorylation, but also controls the activity of other enzymes and the transfer of other PTMs. While numerous large-scale phosphoproteomics studies highlight the extent and diversity of phosphoproteins present in total cell lysates, the further understanding of their regulation and biological activities require a spatio-temporal resolution only achievable through subcellular fractionation. In the emerging field of subcellular phosphoproteomics, cell fractionation approaches can combine with sensitive MS methods to facilitate the identification of low abundance proteins and to unravel the intricate regulation of protein phosphorylation [113].

A global and quantitative analysis of protein phosphorylation provides a powerful new approach and has the potential to reveal new insight in signaling pathways. Recent technological advances in high resolution mass spectrometers and multidimensional LC, combined with the use of stable isotope labeling of proteins, have led to the application of quantitative phosphoproteomics to study *in vivo* signal transduction events on a proteome-wide scale [114–118].

## Figures and Tables

**Table 1:** Diverse functions of post-translational modifications (PTMs) in cancer and in lung cancer.

Types of PTMs	Functions and roles	References
Acetylation	Protein stability, protection of *N* terminus, regulation of protein/DNA interactions (histones)	[18]
Acylation	Cellular localization and targeting signals, membrane tethering, mediator of protein/protein interactions	[19]
Deamidation	Possible regulator of protein/protein and receptor/ligand interactions	[20]
Disulfide-bond formation	Intramolecular and intermolecular crosslink, protein stability	[21]
Glycosylation (*N*-linked, *O*-linked)	Excreted proteins, membrane proteins, cell-cell recognition/signaling *O*-GlcNAc, reversible, regulatory functions	[22]
GPI anchor	Glycosylphosphatidylinositol anchor, membrane tethering of enzymes/receptors	[23]
Methylation	Regulation of gene expression	[24]
Phosphorylation (Tyr and Ser/Thr)	Reversible, activation of enzyme activity, modulation of molecular interactions, signaling	[25]
Nitration of tyrosine	Oxidative damage during inflammation	[26]
Ubiquitination	Signal of degradation	[27]

**Table 2:** While titanium dioxide (TiO_2_) and zirconium oxide (ZrO_2_) mainly elute monophosphorylated peptides, immobilized metal affinity chromatography (IMAC) chiefly elutes multiphosphorylated peptides. The enrichment process consists of several steps which include: incubation (binding), washing to remove non-specific peptides, and elution of phosopeptides. These steps depend on: the buffer, material of solid support as well as the concentration of the sample.

	Mainly *	Phosphoenrichments
**Binding**	Monophosphorylated	TiO_2_ ≈ ZrO_2_ > IMAC
	Multiphosphorylated	TiO_2_ ≥ ZrO_2_ > IMAC
**Eluting**	Monophosphorylated	TiO_2_ ≈ ZrO_2_ > IMAC
	Multiphosphorylated	TiO_2_ ≤ ZrO_2_ < IMAC

* : Depending on the following steps: incubation→washing→elution, take into account: buffer→material of solid support → sample concentration.

**Table 3:** Several combinations of phosphoenrichments (e.g., SCX, SIMAC and Ti^4+^-IMAC Microspheres) make it possible to obtain complementary data and large scale analysis.

Different phosphoenrichment combinations	Binding and eluting with high yield
SIMAC (IMAC coupled to TiO_2_)	Mono and multiphosphorylated
SCX coupled to IMAC and TIO_2_	Mono and multiphosphorylated
SAX coupled to IMAC and TiO_2_	Mono and multiphosphorylated
Ti^4+^-IMAC microspheres	Mono and multiphosphorylated
Calcium phosphate precipitation coupled to IMAC and TiO_2_	Mono and multiphosphorylated

**Table 4:** Critical events during sample preparation prior analyses of phosphorylated proteins *via* proteomics and mass spectrometry.

Cons	Pros
Lyophilization	Phosphatase inhibitors
Salts	Correct adjustment of the peptides sample amount according to the material/beads which captures the phosphopeptides
Detergents	
Phosphate buffers	
